# gga-miRNOME, a microRNA-sequencing dataset from chick embryonic tissues

**DOI:** 10.1038/s41597-022-01126-7

**Published:** 2022-01-31

**Authors:** Isabel Duarte, Gil Carraco, Nayara T. D. de Azevedo, Vladimir Benes, Raquel P. Andrade

**Affiliations:** 1 Faculdade de Medicina e Ciências Biomédicas (FMCB), Universidade do Algarve, Campus de Gambelas, 8005-139 Faro, Portugal; 2 Center for Health Technology and Services Research (CINTESIS), Polo da Universidade do Algarve, 8005-139 Faro, Portugal; 3 ProRegeM-PhD Program in Mechanisms of Disease and Regenerative Medicine, Faro, Portugal; 4 ABC-RI, Algarve Biomedical Center Research Institute, Faro, Portugal; 5 Genomics Core Facility, EMBL, Heidelberg, Germany; 6 Champalimaud Research Program, Champalimaud Center for the Unknown, Lisbon, Portugal

**Keywords:** miRNAs, Non-model organisms

## Abstract

MicroRNAs (miRNAs) are small non-coding RNA molecules, with sizes ranging from 18 to 25 nucleotides, which are key players in gene expression regulation. These molecules play an important role in fine-tuning early vertebrate embryo development. However, there are scarce publicly available miRNA datasets from non-mammal embryos, such as the chicken (*Gallus gallus*), which is a classical model system to study vertebrate embryogenesis. Here, we performed microRNA-sequencing to characterize the early stages of trunk and limb development in the chick embryo. For this, we profiled three chick embryonic tissues, namely, Undetermined Presomitic Mesoderm (PSM_U), Determined Presomitic Mesoderm (PSM_D) and Forelimb Distal Cyclic Domain (DCD). We identified 926 known miRNAs, and 1,141 novel candidate miRNAs, which nearly duplicates the number of *Gallus gallus* entries in the miRBase database. These data will greatly benefit the avian research community, particularly by highlighting new miRNAs potentially involved in the regulation of early vertebrate embryo development, that can be prioritized for further experimental testing.

## Abstract (summary)

**Table:** 

Measurement(s)	miRNA
Technology Type(s)	microRNA profiling assay
Factor Type(s)	embryonic tissue
Sample Characteristic - Organism	Gallus gallus

Machine-accessible metadata file describing the reported data: 10.6084/m9.figshare.17173127

## Background & Summary

MicroRNAs (miRNAs) are small, single-stranded RNAs with sizes ranging from 18 to 25 nucleotides that are involved in gene expression regulation. This is achieved via post-transcriptional silencing of complementary messenger RNA (mRNA) targets by repression of translation and/or mRNA degradation^1^. miRNAs were initially called small temporal RNAs (stRNAs), since they were first described as essential for proper developmental stage transition in the *C. elegans* life cycle^2^. Today they are recognized to act as gatekeepers of developmental time in many other systems, by mediating cell proliferation-to-differentiation transitions^3^.

The canonical pathway of miRNA biogenesis starts with the transcription of a primary miRNA (pri-miRNA) by RNA polymerase II. The pri-miRNA forms a hairpin that is recognized by DGCR8, which recruits a Class 2 ribonuclease III enzyme, Drosha. This enzyme cleaves the RNA releasing the hairpin, called precursor miRNA (pre-miRNA), which is then exported to the cytoplasm via the Exportin-5 transporter. Here, it is recognized by a second Class 2 ribonuclease III enzyme, Dicer, that cleaves the loop from the hairpin releasing a small double-stranded RNA. One of the strands binds to an Argonaute protein from the RNA-induced silencing complex (RISC), while the other is degraded. At this point, the mature miRNA selectively recognizes and binds to the 3′ untranslated region (3′UTR) of its target mRNA through a small 2–7 nucleotide seed region, leading to RISC-mediated mRNA degradation and/or translational repression^1^.

An essential step in addressing miRNA-mediated regulation of gene expression is to identify and quantify the miRNAs present in the biological system of interest. High throughput miRNA profiling studies have identified thousands of miRNAs in Human and mouse samples^4^. However, this effort has been lagging behind in other model organisms, hindering the elucidation of their role in these systems. This is the case of the chicken (*Gallus gallus*) embryo, a well-established model for studying human embryogenesis due to its extraordinary molecular and morphological similarities in the early stages of development, alongside the ease of experimental manipulation it offers^5^. It was in the chicken embryo that the molecular embryonic clock (EC) underlying the periodic formation of vertebrae precursors was first described^6^. EC genes present cyclic expression maintained by negative feedback regulation in the posterior undetermined presomitic mesoderm (PSM), which gradually slows in the anterior PSM and halts in the segmented somites^7,8^. The periodicity of gene expression oscillations in the PSM is species-specific but can also differ in different tissues of the same organism. Namely, *hairy2* gene expression oscillates with a periodicity of 90 min in the chick PSM and 6 h in the distal cyclic domain (DCD) of the developing forelimb bud^9^.

mRNA instability is essential for EC cycles of expression and there is evidence of a miRNA-dependent regulation of EC gene oscillations^10^. Namely, miR-125a-5p is required for cyclic *LFNG* expression in the chick PSM^11^ and miR-9 drives *Hes1* oscillations in mouse neural progenitor cells^12^. Additionally, we previously showed that the genes encoding the enzymatic machinery for miRNA biogenesis are expressed in both the chick PSM and forelimb bud^13^, tissues where the EC is oscillating.

**Fig. 1:**
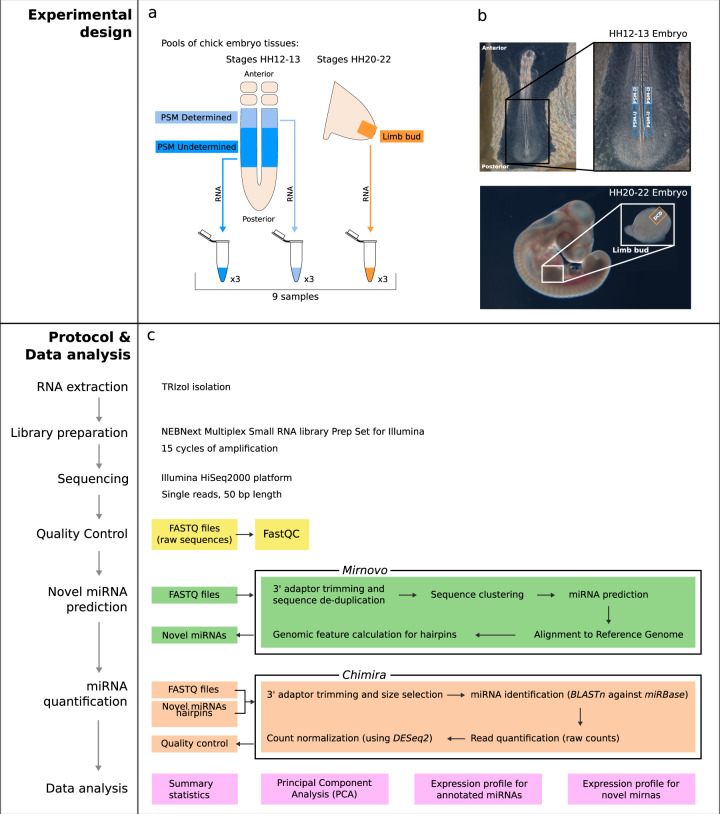
Experimental design, protocol overview, and data analysis workflow. (**a**,**b**) Overview of the experimental design, showing the sampling sites of the chick embryonic tissues collected. (**c**) Pipeline for annotated miRNA-seq data analysis and novel miRNA prediction. PSM_D: determined Presomitic Mesoderm (PSM); PSM_U: undetermined PSM; DCD: Limb Distal Cyclic Domain.

A thorough characterization of the role of small RNAs in chick embryo development and in the regulation of the EC has been hampered by the scarcity of miRNA expression datasets in embryonic tissues of this model system. To overcome this limitation, we performed a miRNA profiling analysis (miRNA-Seq) of three different tissues of the developing chick embryo (Fig. 1a,b). Namely, two regions of the PSM - Undetermined Presomitic Mesoderm (PSM_U) and Determined Presomitic Mesoderm (PSM_D) - and the Forelimb Distal Cyclic Domain (Limb). We report the identification of 926 known miRNAs, and 1,141 candidate novel miRNAs, not previously described in chicken. Accordingly, we believe that this will be an invaluable data resource for the research community studying miRNA-mediated gene expression in early vertebrate development, particularly in the chick embryo.

## Methods

### Embryos

Fertilized *Gallus gallus* eggs (Pintobar, Portugal) were incubated at 38 °C in a humidified atmosphere for two or four days to obtain embryos in stages HH12–13 and HH20–22^14^, respectively.

### Sample collection

Presomitic mesoderm (PSM) tissues were isolated from embryos in stages HH12–13. To obtain these samples, embryos were collected from 48h-incubated eggs, placed in a petri dish containing phosphate buffer saline (PBS) solution and staged according to Hamburger and Hamilton^14^. Only the embryos in stages HH12–13 were selected for further use. The embryos were then placed ventral side up in PBS and 4 μL of Pancreatin (25 mg/mL) (Sigma #8049-47-6) was added to the surface of the embryo. After 3 to 5 minutes, pancreatin was inactivated with goat serum (Gibco #16210-072). The mesoderm located on either side of the neural tube was isolated from all surrounding tissues and divided into determined PSM (PSM_D, upper one-third portion) and undetermined PSM (PSM_U, caudal two-thirds) (Fig. 1a,b). Due to the extraordinarily small size of these tissues, 20 pairs of PSM portions were pooled together for RNA extraction from each biological sample. The samples were snap frozen in liquid nitrogen and stored at -80 °C.

Distal Cyclic Domain (DCD Limb) tissues were isolated from embryos at stages HH20–22 (Fig. 1a,b). Embryos were collected from fertilized eggs incubated for four days, placed in PBS and staged according to Hamburger and Hamilton^14^. Only the embryos in stages HH20–22 were selected for further use. The limb tissue (distal medial portion of the forelimb bud) was manually dissected using forceps. 20 DCD Limb pairs were pooled together for each sample, snap frozen in liquid nitrogen and stored at -80 °C.

### RNA extraction

Biological samples were defrosted on ice. Total RNA was extracted using TRIzol Reagent (Invitrogen #15596-018) according to the manufacturer’s instructions with slight adaptations, namely, the aqueous phase from the first step of extraction was washed once with Phenol:Chloroform (Sigma #P2069) and then with Chloroform:Isoamyl Alcohol (24:1). The aqueous phase was recovered using Phase lock Gel Heavy (5Prime #2302830) and RNA was precipitated by addition of 1/10 volume of 3 M sodium acetate, 2.5 volumes of 100% ethanol and 3 μL per mL of Linear Acrylamide (Ambion #AM9520). After one hour at -80 °C, the RNA was precipitated by centrifugation at 14,000 rpm for 30 minutes at 4 °C. The pellet was washed with 70% ethanol and centrifuged for 15 minutes at 4 °C, briefly air-dried and resuspended in 50 µL of MilliQ (Merck Millipore) purified water. The samples were quantified using NanoDrop 2000 (Thermo Scientific) and stored at -80 °C.

### RNA quality control

A first-round of quality control was performed by Reverse Transcription-PCR. 100 ng of RNA was reverse transcribed using iScript™ cDNA Synthesis Kit (BioRad #1708890). Subsequent PCR for GAPDH was done using DreamTaq DNA Polymerase (Thermo Scientific™ #EP0701). In a second instance, RNA quality control was performed using Experion™ RNA StdSens Analysis Kit (BioRad #700-7103) (Table 1). Only samples with an RQI (RNA Quality Indicator) equal to or above 8.5 were sent for sequencing.

**Table 1:** Total RNA quality control.

Sample ID	Tissue	Hamburger Hamilton Stages (HH)	Concentration (ng/µL)	RQI
Limb1	Forelimb Distal Cyclic Domain	HH20–22	515.72	9.5
Limb2			1,477.60	9.9
Limb3			742.8	9.7
PSM_D1	Determined Presomitic Mesoderm	HH12–13	48.00	9.3
PSM_D2			63.41	10.0
PSM_D3			38.03	9.3
PSM_U1	Undetermined Presomitic Mesoderm	HH12–13	52.10	8.5
PSM_U2			39.24	8.7
PSM_U3			61.50	9.2

### Library preparation and miRNA-sequencing

The sequencing libraries were prepared using the NEBNext Multiplex Small RNA Library Prep Set for Illumina (NEB #E7300S/L Version 5.0), starting with 150 ng of total RNA as input. As a first step in the protocol, adaptors ligate directly to the small RNA fragments containing 5′ phosphate and 3′ OH, followed by cDNA generation and PCR amplification. 15 cycles of amplification were performed using specific SR primers for Illumina and index primer of choice for each sample (according to NEB #E7300S/L Version 5.0 protocol).

Size distribution of the final library was assessed on Bioanalyzer (Agilent Technologies) with a DNA High Sensitivity kit (Agilent Technologies #5067-4626), and concentration was measured with Qubit® DNA High Sensitivity kit (Life Technologies #Q32854) in Qubit® 2.0 Flurometer (Life Technologies). Individual libraries that passed the QC step were pooled equimolarly in a 9-plex, and final pool was purified with SPRI select beads at a 1.3x bead ratio (Beckman Coulter #B23319). Pool was loaded to a single lane of an Illumina HiSeq. 2000 sequencing instrument (Illumina Inc.) at 6 pM concentration and was sequenced in 50 bp single-read mode^15^. Library construction and sequencing were performed at EMBL’s GeneCore facility in Heidelberg, Germany.

### Quality control of sequencing reads

**Fig. 2:**
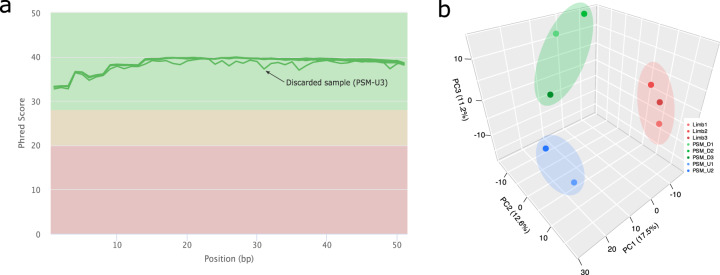
miRNA-seq Quality Control and experimental design validation. (**a**) Raw sequencing reads were evaluated with FastQC^16^. (**b**) Principal Component Analysis (PCA) showing the overall variance between samples.

Sequencing reads were firstly evaluated using FastQC (version 0.11.5)^16^ to verify the overall read quality of each sample. One library (PSM_U2), from undetermined PSM, did not pass the quality control step, mainly due to its small library size, leading to its removal from further analyses (Fig. 2a).

### Quantification and normalization of annotated miRNAs

For the remaining 8 samples that passed the quality control, annotated microRNA read counts were obtained using the Chimira software (version 1.5)^17^. Briefly, the pipeline implemented in Chimira for miRNA-seq analysis comprises the following steps: firstly, the sequences are cleaned, trimmed, and size selected to remove adapters and low quality microRNA reads. Next, the reads passing the previous filters are mapped to *Gallus gallus* hairpin sequences present in miRBase (release 22)^4^ using BLASTn^18^ allowing up to two mismatches. Finally, a count-based miRNA expression dataset is generated^19^ and normalized across all samples using DESeq2^20^. Further data validation, visualization, and statistical analyses were conducted using the normalized log2 expression data.

### Detection, quantification and normalization of novel miRNAs

Detection of novel miRNAs was performed using the Mirnovo tool (v1.0)^21^, which is a machine learning algorithm that predicts novel miRNAs by analysing structural features of miRNA precursor hairpin sequences gathered directly from small RNA-Sequencing data. Briefly, the Mirnovo pipeline entails the following steps: (i) adapter removal followed by sequence de-duplication; (ii) the tallied sequences then enter a series of clustering steps, followed by cluster refinement to obtain consensus sequences; (iii) the prediction step identifies known and novel miRNAs; and (iv) the final step, aligns the consensus sequences from all miRNAs (known and novel) to the reference genome. This was done by selecting the most stable hairpins (scored by Delta G free energy) found in a 90-nucleotide window around the consensus sequences followed by genomic feature calculation^21^.

The specific parameters used for Mirnovo were: *Gallus gallus* input species, using the Universal prediction model (since there are no models specifically trained for chicken), length filter between 16 and 28 nucleotides, minimum read depth of 5, minimum variants 1, and initial clustering using an alignment identity threshold of 0.9 (vsearch-id parameter).

The candidate novel miRNAs were then quantified and normalized using Chimira^17^ with the Mirnovo extension. The analysis was performed as described above for the annotated miRNAs, with the difference that the custom hairpin FASTA files output from Mirnovo for each sample were uploaded together with the corresponding raw FASTQ files.

Data resulting from this identification and quantification (i.e. hairpin sequences, genomic location, and normalized counts) is freely available^22^.

### microRNA expression profiling

Using customized R scripts (R version 3.6)^23^, we conducted quality control analysis, and briefly inspected the profile of annotated and novel microRNA expression in each embryo tissue. For this we used R packages for data visualization, namely, *Tidyverse*^24^, *UpSetR*^25^, *Patchwork*^26^, and *plot3D*^27^.

## Data Records

All sequencing data has been deposited in the ArrayExpress data repository^28^ with accession number E-MTAB-8176^15^. This dataset consists of 8 microRNA expression raw data files in fastq format. Detailed experimental procedures and data analysis are also available there.

Processed data (in tabular text format) containing the log2 normalized counts of the sequencing reads for annotated miRNAs has been deposited in Figshare^19^. Similarly, the list of predicted novel miRNAs, with sequence, and log2 normalized counts is available in Figshare^22^.

All the sequencing data and the normalized miRNA expression counts are open. The R code used for the exploratory data analysis and visualizations are also freely available for consultation in Figshare^29^.

## Technical Validation

### Quality control of microRNA-Seq data

The quality control of the raw sequencing reads was performed using FastQC^16^ to assess overall read quality and flag potentially poor-quality samples. All samples except one, passed the QC metrics performed by FastQC^16^. The poor-quality sample presented a variable PHRED score distribution across the read length (Fig. 2a), as well as a very low total number of reads (244,296 reads compared to 3 million average reads in the other samples), hinting that the sequencing step was faulty, possibly due to sample degradation prior to or during library preparation. Accordingly, this sample was removed from further data analyses.

Chimira, the software used to quantify the miRNA expression, also performs quality control for the samples, namely read length distribution after trimming, nucleotide distribution per position, and GC content ratio at each position. These were all manually inspected (to ensure that biases and outlier sequences were not present) before accepting the output miRNA quantification values.

### Validation of experimental design strategy

Since each tissue sample included pools of 20 embryos, expression variation was expected between the biological replicates for the same tissue. Accordingly, to validate our experimental design and check for sample coherence between replicates, we evaluated sample variance via a Principal Component Analysis (PCA).

The first and second components (17.5% and 12.6% explained variance, respectively) can only distinguish between the Limb and the PSM, but by adding the third component (11.2%) the distinction between determined and undetermined PSM becomes apparent (Fig. 2b). This shows, as expected, that the differences between Limb and PSM, two distinct tissues, are more extensive than the differences between determined and undetermined PSM, two molecular states of the same tissue. Albeit more subtle, such differences within the PSM are visible in the dataset, therefore validating the samples collected for our experimental design (Fig. 1a).

### Performance measures for novel miRNA predictions

**Fig. 3:**
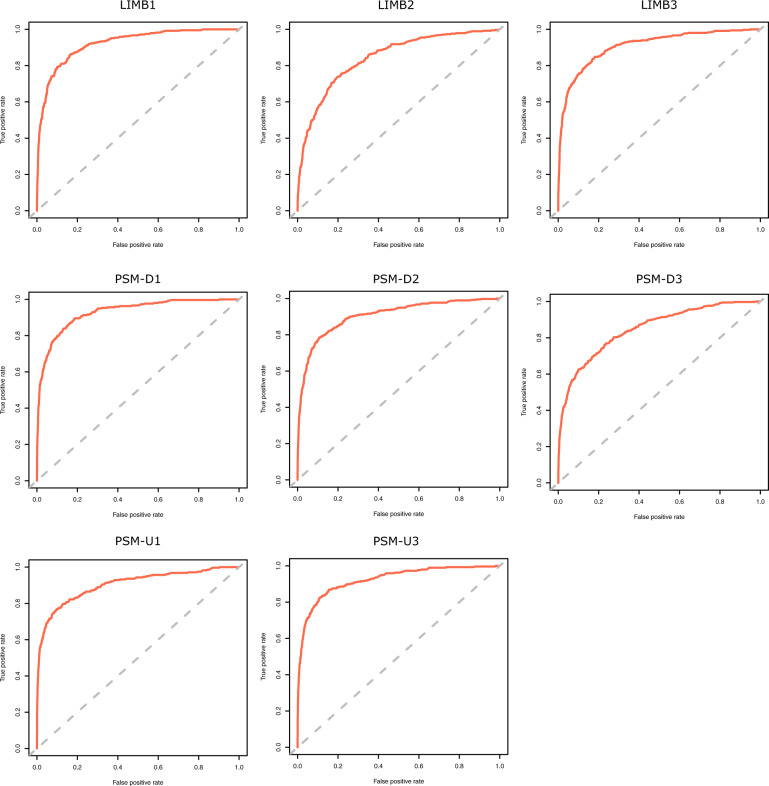
Novel miRNA prediction quality. ROC curves for the random forest algorithm applied to each tissue sample.

The quality metrics reported by Mirnovo for the novel miRNAs predicted for *Gallus gallus* show an overall good scoring for all samples, as seen in the ROC curves reported for the Random Forest algorithm applied (Fig. 3).

As shown in Table 2, the method is highly specific (>95% of true negative identification), despite not being very sensitive (circa 50% true positive identification). This means that although many new miRNAs might be missed, the ones reported should be regarded as highly reliable. These results mirror the fact that the prediction had to be run using a general animal model, given the lack of specific models trained with chicken miRNAs.

**Table 2:** Performance measures from novel miRNA prediction using the Mirnovo algorithm.

Sample ID	Tissue	Performance Measures			Predicted miRNAs
		Sensitivity (%) TP/P	Specificity (%) TN/N	Precision (%)* TP/(TP + FP)	Novel predictions (%) FP/(TP + FP)
Limb1	Forelimb Distal Cyclic Domain	59.18	96.35	52.25	47.75
Limb2		44.06	95.00	44.53	55.47
Limb3		58.40	96.40	55.35	44.65
PSM_D1	Determined Presomitic Mesoderm	59.40	97.24	52.21	47.79
PSM_D2		52.03	97.19	52.56	47.44
PSM_D3		36.79	98.21	39.12	60.88
PSM_U1	Undetermined Presomitic Mesoderm	52.31	98.80	49.33	50.67
PSM_U3		54.36	98.10	47.23	52.77

*The precision metric reported by Mirnovo represents the proportion of known miRNAs (annotated in miRBAse) relative to the total known (True Positives) plus novel predicted (False Positives)^21^.

This method identified circa 50% novel candidate miRNAs as shown by the novel prediction values presented in Table 2. This represents the addition of 1,141 new candidate miRNAs to the previously existing 1,232 mature miRNAs in the miRBase database for *Gallus gallus*, further granting relevance to this dataset.

### Validation of expression profiling

To validate the read normalization and quantification steps, we evaluated the read distribution before and after normalization, and briefly compared the miRNA expression profile between the three tissues. Known and novel miRNA datasets were independently evaluated, since the analysis was conducted separately, and each dataset represents a different resource for the community.

### miRNA-seq Reads distribution and normalization

**Fig. 4:**
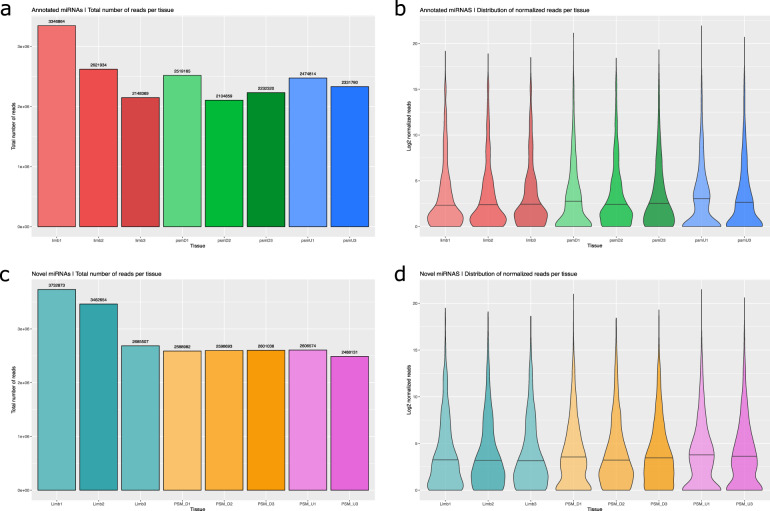
Total counts and distribution of normalized expression per tissue, for annotated (a-b) and novel (c-d) miRNAs. Total number of reads for (**a**) annotated miRNAs, and (**c**) novel predicted miRNAs. Distribution of normalized read counts per tissue, in (**b**) known, and (**d**) novel miRNAs.

The distribution of the total number of reads (Fig. 4a,c) shows that there are some differences between the replicates before read count normalization, particularly for the Determined PSM tissue in the known miRNAs set. This fact is most likely a reflection of the embryo pooling strategy that might be contributing asymmetrically to the total miRNA amount present in each sample. After normalization (Fig. 4b,d), the distributions become more balanced between replicas, and therefore amenable for further expression comparisons between tissues. As expected, the miRNA expression distribution for all three tissues is positively skewed (even after log2 transformation), showing a long tail to the right.

### miRNA Expression profile in the different tissues

Looking at the miRNA expression profile in the three tissues helps with uncovering possible experimental errors; for example, large asymmetries in the diversity of miRNAs found for each tissue could indicate faulty sequencing, or a total overlap of miRNA identities between tissues could indicate mislabelling or inadequate experimental design.

**Fig. 5:**
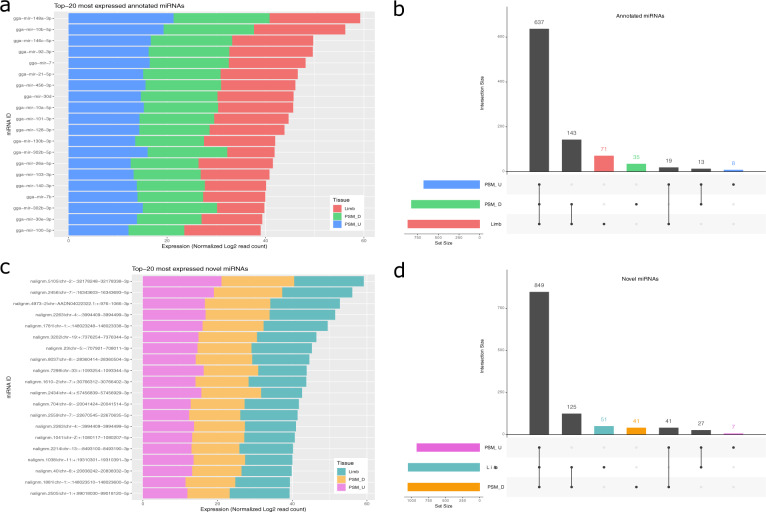
Expression profiling and overlap between the three tissues for annotated (**a**,**b**) and novel (**c**,**d**) miRNAs. (**a**,**c**) Top 20 highly expressed miRNAs per tissue. (**b**,**d**) Intersection between miRNAs expressed in each tissue.

The top-20 most expressed miRNAs (Fig. 5a,c) are found in all three tissues, with roughly comparable distributions in both annotated and novel miRNAs. Additionally, the intersection plot (Fig. 5b,d) clearly shows that the majority of miRNAs (637 in known miRNAs and 849 in novel miRNAs) are found in all three tissues. Importantly, each tissue presents exclusive miRNAs, namely 71 in Limb, 35 in determined PSM, and 8 in undetermined PSM for known miRNAs (Fig. 5b); and 51 in Limb, 41 in determined PSM, and 7 in undetermined PSM for novel miRNAs (Fig. 5d), showing that each sample is sufficiently different from the others, allowing for proper differentiation between tissues.

## Usage Notes

The bioinformatics analysis described here made use of freely available software tools commonly used by the research community (Fig. 1c). There are alternative miRNA-seq analysis pipelines equally applicable to the FASTQ reads from *Gallus gallus*^22^, for example, miRDeep2^30^, QuickMIRSeq^31^. and sRNAnalyzer^32^. For a recently published miRNA-seq analysis protocol, see Potla *et al*.^33^, discussing available individual tools for each step: (i) quality control (adaptor trimming, read quality/length filtering); (ii) read mapping; (iii) annotation (using miRBase); (iv) quantification; and optionally (v) detection of novel miRNAs.

These data can equally be used to seek the complete small RNA’ome, using for example the recently developed platform coMpSRA that is reported to identify and quantify diverse RNA molecule types, including miRNA, piRNA, snRNA, snoRNA, tRNA, and circRNA^34^.

The miRNA expression data herein reported^19^ will be useful to study gene regulation in the early phases of vertebrate embryo development, for example by performing differential expression and target gene annotation analyses. Some considerations should be taken into account for downstream analyses. Namely, the RNA was extracted from pools of 20 dissected tissues meaning that each sample represents an heterogenous mixture of individuals, whose variability is present in the data. This is even more relevant if we consider that oscillations of clock gene expression occur in the tissues analysed. Thus, care should be taken when using such static sample datasets to contrast tissues with dynamical gene expression. Additionally, some SNPs can potentially interfere with the successful mapping of some miRNA transcripts that might have been discarded, and therefore cause an underrepresentation of expression for those miRNAs. Finally, for differential expression studies comprising the PSM_U tissue, since this group comprises only two replicates, the comparison will have lower statistical power to detect small effect sizes. Accordingly, appropriate statistical techniques should be applied to deal with this limitation.

Since the chicken genome annotation is not yet up-to-par with the annotations from other vertebrate genomes, most chicken miRNAs deposited in databases are not yet experimentally validated, and their target genes are based mostly on chicken-specific computational predictions. This study opens the door for new findings specific for birds, and for validation of known vertebrate miRNAs and their respective target genes. Finally, the predicted novel miRNAs^22^ represent an invaluable resource for the avian research community looking to experimentally validate novel candidate miRNAs acting in early vertebrate development capable of regulating their gene of interest. Additionally, these data coupled with transcriptomics data for the same tissues can help uncover potential regulatory modules active in early vertebrate embryogenesis.

## Data Availability

Technical validation and data visualization was performed in RStudio (Version 1.1.463)^35^, using R (version 3.6)^23^, and Bioconductor (version 3.9)^36^, with packages tidyverse (version 1.3.1)^24^, UpSetR (version 1.4.0)^25^, patchwork (version 1.1.1)^26^, plot3D (1.3)^27^. The R code used for these analyses, in the form of an annotated R notebook, is freely available in Figshare^19^. Additional software tools used to analyse this miRNA-seq dataset were the following: FastQC (version 0.11.5)^16^, Chimira (version 1.5)^17^, and Mirnovo (version 1.0)^21^ as described in the Methods section.
